# Energy labelling of alcoholic drinks: An important or inconsequential obesity policy?

**DOI:** 10.1002/osp4.638

**Published:** 2022-09-23

**Authors:** Eric Robinson, Emma Boyland, Rebecca Evans, Amy Finlay, Lauren Halsall, Gabrielle Humphreys, Tess Langfield, India McFarland‐Lesser, Zina Patel, Andrew Jones

**Affiliations:** ^1^ Department of Psychology Eleanor Rathbone Building University of Liverpool Liverpool UK

**Keywords:** alcohol, beverage, calories, drink, energy labelling, nutritional labelling, obesity

## Abstract

Alcohol is calorie dense, but unlike food products, alcoholic drinks tend to be exempt from nutritional labelling laws that require energy content information to be displayed on packaging or at point of purchase. This review provides a perspective on the likely efficacy of alcoholic drink energy labelling as a public health policy to reduce obesity and discusses key questions to be addressed by future research. First, the contribution that alcohol makes to population level daily energy intake and obesity is outlined. Next, consumer need for alcohol energy labelling and the potential impacts on both consumer and industry behavior are discussed. Pathways and mechanisms by which energy labelling of alcoholic drinks could reduce obesity are considered, as well as possible unintended consequences of alcoholic drink energy labelling. Would widespread energy labelling of alcoholic drinks reduce obesity? The unclear effect that alcohol has on population level obesity, the modest contribution calories from alcohol make to daily energy intake and limited impact nutritional labelling policies tend to have on behavior, suggest alcohol energy labelling may have limited impact on population obesity prevalence as a standalone policy. However, there are a number of questions that will need to be answered by future research to make definitive conclusions on the potential for alcohol energy labelling policies to reduce obesity.

## 1 BACKGROUND

Increases to population level energy intake have been identified as a key driver of the rise in overweight and obesity observed across most of the developed world during the 20th century.^1^ The global obesity epidemic has a substantial public health burden and is likely to be responsible for more than 2 million deaths per year.^2^ The wide availability of calorie dense food and drink products has been identified as a contributor to excess energy intake.^1^, ^3^, ^4^, ^5^ One common policy approach used to improve population level diet is nutritional labelling.^6^ For the majority of commercially produced and packaged food products sold in the UK, EU and elsewhere,^7^, ^8^, ^9^ it is required by law that products provide on‐label nutritional information, such as energy content.^10^ Food products sold in the out‐of‐home food sector, such as restaurants and fast‐food outlets, have traditionally been exempt from these rules (e.g., on menus). However, a number of countries including the US, England and parts of Canada and Australia,^11^, ^12^ have passed laws that require large out‐of‐home food sector (OOHFS) businesses to provide customers with energy information at point of purchase. Energy labelling in the OOHFS has been made mandatory on the basis that energy information allows consumers to make better informed food choices and therefore has potential to reduce population level obesity.^12^

Alcohol is energy dense; a single gram of alcohol contains ∼7 kcals, making it higher in energy density than both protein and carbohydrate (4 kcals/g) and second to fat, the nutrient with the highest energy density of 9 kcals/g.^13^ Current recommended average daily energy intake for women and men is 2000 and 2500 kcal, respectively.^14^ The number of calories in alcoholic drinks varies and can be substantial; a 355 ml serving of beer can range from 103 to 250 kcals dependent on alcoholic strength and brand, whereas wines and cocktails can range from below 100 kcals to >500 kcals dependent on serving size, alcoholic strength and other ingredients.^15^ In addition to non‐negligible amounts of macronutrients (e.g., carbohydrates), alcohol content tends to account for the most significant proportion of the energy content of alcoholic drink products.^15^ Although alcoholic drink products are widely advertised, sold and consumed both in and outside of the home,^16^ products containing an alcohol by volume (ABV) of >1.2% (the majority of beers, wines, spirits and other widely consumed alcoholic products) tend to be exempt from laws that require nutritional information on product labels or at point of purchase in most countries.^17^, ^18^, ^19^ Indeed, there are stricter labelling requirements for bottled water compared to alcohol, across Europe.^20^

In the US, energy labelling is required for alcoholic drinks sold by large businesses in the OOHFS, but this is not the case in other countries with OOHFS energy labelling laws, such as England. In the absence of mandatory laws that require energy labelling of alcoholic drinks there is very limited voluntary provision of this information, as evidenced by recent EU and UK studies.^17^, ^21^ In a 2017 report, the World Health Organization (WHO) concluded that ‘providing consumers with information about the calorie content of alcoholic beverages is a potentially important way of helping them reduce their calorie intake if they so wish’^22^ and a 2017 European Commission report suggested ‘there were no objective grounds that justify the absence of nutritional information from alcohol products.’^17^ Because the alcohol industry have tended not to follow these recommendations and provide nutritional information voluntarily, there are now calls for mandatory nutritional labelling of alcoholic drinks. For example, Ireland is the first EU member state to recently pass legislation that will require alcoholic drink packaging to include energy content information.^17^ Similarly, since leaving the EU, UK government has announced an intention to consider making energy labelling of alcoholic drinks mandatory by law in England, though they are yet to formally introduce legislation.^23^

In the present article a critical perspective on alcohol energy labelling as a public health policy to reduce obesity is provided, as alcohol energy labelling legislation has been framed by the WHO and more recently by the UK government as a policy to reduce energy intake and obesity.^22^, ^23^ Although beyond the scope of this review, given the sizable disease burden that alcohol consumption directly causes,^24^ policies which successfully reduce alcohol consumption should also lead to direct health benefits irrespective of effects on body weight. In countries with extensive mandatory policies for energy labelling of food (e.g., US, England), energy labelling is required on both packaged food and for products sold in the OOHFS, whereas more complete nutrition information (e.g., ingredient list, full macronutrient composition per 100 g) is only required on pre‐packaged food products. For this reason, where possible research specifically relating to energy labelling is drawn on, as opposed to more extensive nutritional labelling. Although not a systematic literature review or formal literature review, electronic database searches were conducted to identify relevant research (see online Supplementary Materials).

Moving beyond a recent rapid systematic review that focused only on consumer understanding of the energy content of alcoholic drinks, support for and individual consumer behavior in response to alcohol energy labelling,^25^ the likely impact that alcohol consumption has on population level energy intake and obesity is considered (*Alcohol: contributions to energy intake and obesity*), as this will be key to effectiveness of alcohol energy labelling policies. Next, a perspective on whether there is a genuine consumer need for energy labelling of alcohol drinks is discussed (*Consumer need for mandatory energy labelling of alcoholic drinks*). The potential impact that mandatory energy labelling policies would have on individual consumer behavior (*Consumer responses to energy labelling*), potential for unintended consequences (*Potential unintended consequences*) and impacts on the behavior of the alcohol industry (*The impact of mandatory energy labelling on the alcohol industry*) are considered. Finally, future research directions are discussed.

## 2 ALCOHOL: CONTRIBUTIONS TO ENERGY INTAKE AND OBESITY

The size of contribution to overall energy intake that alcohol has will vary based on social and cultural differences in drinking frequency and dietary patterns. In England, approximately half of adults drink alcohol on a weekly or more frequent basis.^26^ Using data from a nationally representative survey of English adults in 2000/2001, Gibson & Shirreffs^27^ estimated that alcoholic drinks make a small but significant contribution to daily energy intake equating to approximately 7% and further data collected in 2008/2009 from English adults produced similar results.^28^ Nationally representative data from both the US,^29^ Canada^30^ and Australia^31^ indicate that energy from alcohol also makes a small but significant contribution to population level energy intake. For example, national data from the US (2007–2010) suggests that on average US adults consume 100 kcals per day from alcohol.^32^ Yet, because population sub‐groups vary in their alcohol consumption, with women, younger adults and some ethnic groups being more likely to consume little or no alcohol,^33^ the size of contribution alcohol makes toward daily energy intake across populations will vary substantially and make only a very small contribution in a significant proportion of people.

Because studies tend to indicate that energy intake from alcohol is not fully compensated for (e.g., by eating less food),^34^, ^35^ energy consumed from alcohol is likely to make a small contribution to population level energy intake in countries where individuals drink regularly. As well as directly contributing to daily energy intake due to the energy content of alcoholic beverages, alcohol consumption may also indirectly affect energy intake through the psychological or pharmacological effects ethanol has on appetite stimulation.^34^, ^36^ In line with this, a recent systematic review and meta‐analysis of short‐term experimental studies conducted in laboratory settings^35^ found that acute administration of alcohol (relative to a placebo drink) increased subsequent food intake by 82 kcals. As well as promoting increased energy intake, there is also some evidence to suggest that alcohol may affect how energy is stored in the body. Laboratory studies indicate that alcohol served with food suppresses the rate of fat oxidation and this process likely increases the deposition and storage of body fat, compared to when food is consumed in the absence of alcohol.^37^, ^38^

Although there are both plausible direct and indirect pathways by which alcohol contributes to population level energy intake and therefore may increase risk of overweight and obesity, results of epidemiological studies examining the relationships between alcohol consumption and adiposity have produced mixed findings.^39^, ^40^, ^41^ A recent systematic review and meta‐analysis consisting of 127 studies aimed to overcome some of the inconsistencies in results observed across previous studies.^39^ Pooled analyses of cross‐sectional studies demonstrated that higher alcohol intake was associated with increased odds of abdominal obesity (OR = 1.19 [95% CI: 1.09–1.29]) and having overweight or obesity based on body mass index (BMI) (OR = 1.23 [95% CI: 1.11–1.37]), but not obesity independently (BMI ≥30 kg/m^2^). There was also some evidence of a significant dose‐dependent response between alcohol intake and adiposity when heavier drinking patterns (defined within individual studies) were compared to no or light alcohol drinking across studies.^39^ However, a meta‐analysis of a smaller number of longitudinal studies reviewed found no significant association between alcohol drinking and risk of overweight or obesity,^39^ which raises doubt on the assumption that alcohol drinking drives population level obesity.

One explanation offered for these mixed findings is differences in drinking habits between sampled populations, such that samples characterized by low levels of alcohol drinking obscure any relationship between alcohol consumption and adiposity. In line with this reasoning, it has been suggested that heavier alcohol drinking patterns are more consistently associated with weight gain and heavier body weight in empirical studies that do show an association between alcohol drinking and heavier body weight.^40^, ^42^, ^43^ Some of the inconsistent associations between alcohol intake and adiposity may also be in part explained by studies failing to account for non‐linear relationships; a number of recent studies suggest that light drinkers may have a reduced risk of obesity or a lower BMI compared to non‐drinkers.^44^, ^45^ Interpretation of results is difficult but may in part be explained by non‐drinkers suffering from ill health (and thus abstaining from alcohol) or light drinkers being more likely to engage in healthier lifestyle behaviors, such as frequent physical activity, as opposed to small amounts of alcohol preventing weight gain.^40^ Studies have also tended to rely on self‐reported alcohol consumption, and have differed in how the frequency of drinking (e.g., 24‐h recall, food diaries), type of drinks consumed, and calorie contribution of drinks (e.g., the drink as a whole vs. the pure alcohol component) have been measured and/or accounted for in analyses. All of these factors have been offered as further explanations for mixed findings to date.^38^, ^40^ These limitations aside and inconsistent results aside, there is some consensus that a more frequent and heavier pattern of alcohol consumption is a likely risk factor for increased risk of adiposity.^39^, ^40^, ^41^, ^46^ This may be of particular relevance to college/university aged young adults, as the adoption of frequent and heavier drinking patterns (as opposed to abstinence) in this age group may be a cause of early adult life weight gain.^47^

Given that evidence shows: (i) population level energy intake is responsible for overweight and obesity prevalence, (ii) alcohol makes a significant contribution to energy intake in population groups that regularly consume alcohol, and (iii) there is some tentative evidence linking greater alcohol consumption with risk of obesity, public health approaches that address the amount of energy consumed from alcohol have potential to reduce obesity. However, because the contribution that alcohol makes toward daily energy intake across the population is likely to be relatively small and there is a lack of clear and convincing evidence for alcohol consumption being a significant causal contributor to weight gain and obesity, substantial uncertainty surrounds the size of impact that alcohol related policies would have on obesity.

## 3 CONSUMER NEED FOR MANDATORY ENERGY LABELLING OF ALCOHOLIC DRINKS

An auditing study of alcoholic drinks sold in supermarkets in 15 EU member states during 2013 found that less than 5% of products sampled presented on‐label energy information.^48^ Similarly, a 2014 UK study found only 1% of examined alcoholic drink products in supermarkets provided energy information on labels.^21^ Since 2014, there appears to have been an increase in energy labelling of alcoholic drink products sold in UK supermarkets,^18^ although a 2020 study of supermarket sold alcoholic drinks still found that the majority (63%) of products sampled contained no energy information on product labels.^18^ There are no auditing studies of energy labelling of alcoholic drinks sold in the out of home sector (e.g., pubs, bars and restaurants) in the UK, but this practice is uncommon. In line with this, a 2021 study of packaged alcoholic beverages on sale in Victoria, Australia found that 80% of sampled alcoholic beverage packaging did not contain any nutritional information.^49^ Although energy labelling is rare, the extent to which there is a consumer need is also dependent on whether people are aware of the number of calories in alcoholic drinks and the extent to which consumers believe they should be provided with this information.

A number of studies have examined whether or not people are accurate when asked how many calories there are in common alcoholic drinks.^50^, ^51^ A recent systematic review and meta‐analysis of eight studies that sampled participants from across Europe, North America and Australasia found that approximately 74% of sampled participants were unable to identify the number of calories in common alcoholic drinks accurately.^25^ The review concluded there was moderate evidence that most people are unaware of the energy content of common alcoholic drinks, due to there being a relatively small number of included studies. A subsequent UK study produced similar results; most participants were inaccurate when estimating energy content of common alcoholic drinks with between 76% and 87% of participants making incorrect estimates, dependent on drink type.^52^ In the same systematic review,^25^ studies examining consumer support for energy labelling of alcoholic drinks were examined.^53^, ^54^ Pooled across studies sampling participants from countries in Europe, North America and Australasia, 64% of participants supported the introduction of energy labelling on alcoholic drinks and in each study, participants were more likely to support than oppose the introduction of energy labelling for alcoholic drinks.

Alcoholic drink energy labelling is rare and energy content of drinks can be highly variable. As the majority of consumers are largely unaware of the energy content of alcoholic drinks and tend to believe that energy labelling should be provided on alcoholic drinks, this suggests a clear consumer need for energy labelling of alcoholic drinks.

## 4 IMPACT AND POTENTIAL EFFECTIVENESS OF ENERGY LABELLING

For energy labelling of alcoholic drinks to reduce obesity prevalence, its introduction would need to alter population level energy balance (e.g., by reducing total daily energy intakes or increasing energy expended). There are two main pathways by which labelling policies could affect energy balance; directly by causing individuals to change their behavior (e.g., choosing lower energy drink options or increasing physical activity) and/or indirectly by altering the behavior of the alcohol industry (e.g., reformulating and developing alcoholic drinks to have a lower energy content).

## 5 CONSUMER RESPONSES TO ENERGY LABELLING

**FIGURE 1:**
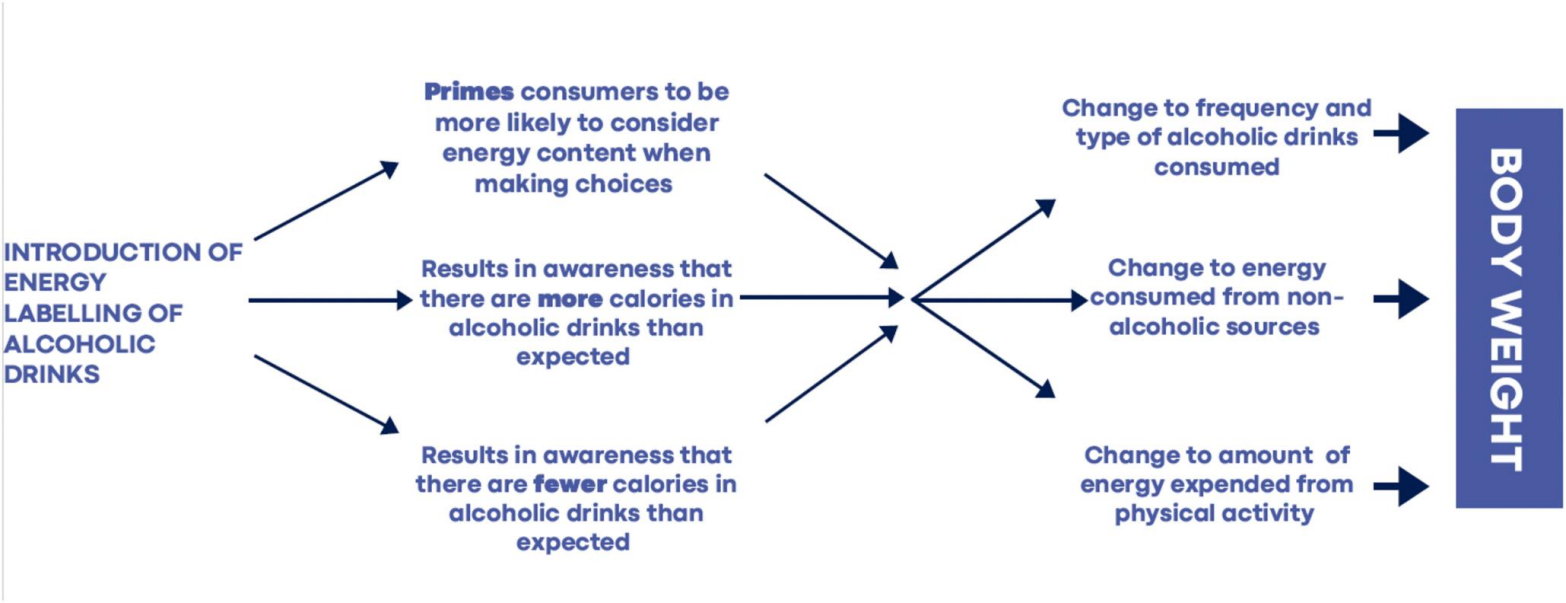
Schematic of processes by which alcohol energy labelling may impact on body weight via individual consumer behavior

Figure 1 outlines some of the direct pathways and psychological mechanisms by which energy labelling of alcoholic drinks could alter individual consumer behavior. First, energy labelling could serve to ‘prime’^55^ or remind consumers that alcoholic drinks contain calories and in doing result in their behavior being more strongly influenced by energy balance considerations. Theoretical models of priming predict that effects on behavior would primarily be observed among those who are already calorie conscious, as primes serve to activate existing goals or intentions (e.g., avoiding consuming too many calories). There is evidence that priming cues or reminders about calories or healthy eating result in some people eating less^56^ and in line with theoretical predictions these effects tend to be observed only among weight conscious individuals.^56^ Although untested in relation to energy labelling of alcoholic drinks, the processes may work in the same way with labelling resulting in individuals making more weight conscious decisions on what and/or how much to drink.

An alternative but not mutually exclusive possibility is that alcoholic drink labelling may serve to correct inaccurate misperceptions of drink energy content. Because a substantial proportion of people tend to underestimate the number of calories in alcoholic drinks,^25^, ^52^ labelling may correct this misperception and result in those individuals choosing a lower energy option. However, a significant number of participants in studies examining knowledge of drink energy content also overestimate and believe there are more calories in drinks than in reality.^25^, ^52^ In such instances labelling could result in consumers becoming aware there are fewer calories in drinks that what they previously believed, which based on the same reasoning could result in a form of licensing effect whereby consumers feel vindicated to drink more or select a higher energy option.^57^, ^58^ In the context of alcohol energy labelling, these proposed mechanisms have not been tested, but there is evidence from the food literature that the impact energy labelling has on consumer behavior is most likely to occur when there is a lack of congruency (or mismatch) between perceived and actual energy content of food products.^57^

Although energy labelling may increase consumer knowledge of the calorie content of food and drink products,^59^ there is limited evidence testing the effect of energy labelling on alcoholic drinks on energy balance related behaviors. A systematic review of six experimental studies examining the effect of labelling on alcohol drinking‐related outcomes concluded that evidence did not suggest that labelling affected consumer behavior, but due to the very low methodological quality of studies this conclusion was uncertain.^25^ Furthermore, studies tended to use self‐reported hypothetical drinking behavior as outcome measures and no studies were conducted in real‐world settings. Since this review, a randomised control trial of UK adults that examined intentions to limit alcohol drinking in response to either being exposed to drink labels with or without energy information found that participants were more likely to report intending to drink less when exposed to energy information on labels.^52^ Although alcohol drinking intentions do tend to be a good predictor of drinking behavior,^60^ there is a well‐observed ‘intention‐behavior’ gap and the use of intended drinking is a limitation of this study. Therefore, the study provides some initial evidence that energy labelling may alter drinking behavior.

A caveat of studies conducted to date is that energy labelling tested has tended to provide kcal information alone (e.g., kcals per serving) and other ways of presenting nutritional information (e.g., physical activity equivalence information) may promote greater attention and consumer understanding.^61^ All experimental studies to date have focused on the impacts of labelling on alcoholic drink choice and behavior, although, as depicted in Figure 1, energy labelling could also theoretically result in changes to what individuals choose to eat (through compensation, e.g., eating less to account for calories from alcohol) or how much energy they expend (e.g., engaging in physical activity to compensate for calories consumed from alcohol). Even relatively small changes to dietary patterns or physical activity levels can prevent weight gain.^62^ Furthermore, these pathways will be particularly important to understand because as discussed, alcohol makes a relatively modest direct contribution to daily energy intake. The majority of daily energy intake is derived from meals and snacks^29^ and therefore energy labelling driven changes to diet may have a more substantial effect on obesity than changes to alcohol consumption alone. A small number of survey studies do suggest that a minority of individuals report they would be likely to eat more healthily or exercise more if energy labelling was provided on alcoholic drinks,^52^, ^63^ but real‐world testing of the impact of alcohol energy labelling on eating or physical activity related outcomes to date have been limited.

Although there is a relative absence of evidence on the impact that alcoholic drink energy labelling has on real‐world consumer behavior, there have been a number of systematic reviews and meta‐analyses examining the impact of food energy labelling on consumer behavior in restaurants, canteens and fast food outlets.^12^ Consistent with suggestions that health information provision‐based interventions (e.g., listing energy content) that rely on conscious effort and motivation on the part of the consumer tend to have relatively modest effects on behavior,^64^, ^65^ reviews to date have concluded that energy labelling of food menus has either no effect on behavior or results in only a small decrease to total calories ordered.^12^, ^59^, ^66^, ^67^ For example, a 2018 Cochrane review and meta‐analysis concluded, on the basis of three low quality studies, that energy labelling resulted in 47 kcals less being purchased per meal.^67^ Whilst a more recent US study examining the introduction of energy labelling in a fast food chain observed an initial reduction of 60 kcals per transaction immediately after introduction, over the course of the next year this reduced to 23 kcals per transaction.^68^ Given that the energy content of meals in restaurants and fast‐food chains is high, commonly exceeding 900 kcals,^5^, ^69^ such changes would equate to reductions in energy ordered of ∼2–5%. Any effects of alcoholic drink energy labelling on consumer behavior may be similarly small in size and be smaller in absolute terms, as total energy consumed from alcoholic drinks is considerably smaller than the energy consumed from food sources.

As more evidence emerges on the impact that energy labelling of alcoholic drinks has on behavior, the extent to which these impacts are observed consistently across different sub‐groups of the population will be important to examine. There have been suggestions that health information provision‐based interventions, such as energy labelling may be less effective among lower socioeconomic positioned (SEP) groups compared to higher SEP groups.^70^ Although there is limited direct evidence in support of this in the food labelling literature,^71^ because lower SEP is associated with a reduced tendency to be motivated by weight control when making dietary decisions and such motives may determine whether or not energy labelling affects consumer behavior,^72^ it is plausible that energy labelling may alter consumer behavior of higher SEP groups more so than lower SEP. The one study that has found evidence that energy labelling of alcoholic drinks increases intentions to reduce alcohol drinking did not find any evidence to suggest results differed by SEP,^73^ but further testing in the real world is needed.

Drinking frequency may also be important to consider, because presumably those who drink more frequently would be more likely to be exposed to energy labelling, although may also be less prone to change their behavior. Similarly, because weight control motives are more common in females and those with overweight and obesity,^74^, ^75^ it is plausible that energy labelling of alcoholic drinks would be less impactful among males with ‘normal’ or healthy weight and future research examining the impacts of alcoholic drink energy labelling will benefit from examining how equitable the effects on consumer behavior are. In particular, because both obesity and alcohol‐related harm are more pronounced in lower SEP groups,^76^, ^77^ future research will need to examine whether the effects of alcoholic drink energy labelling policies are equitable or may serve to widen socioeconomic health‐based inequalities.

## 6 POTENTIAL UNINTENDED CONSEQUENCES

Because there are a limited number of studies examining the specific impact that energy labelling of alcoholic drinks has on consumers there is currently no convincing evidence that energy labelling of alcoholic drinks would produce unintended or undesirable consequences for physical health or well‐being. However, research from studies examining energy labelling of food products and existing observational studies of whether people report compensating for the energy content of alcohol by engaging in ‘unhealthy’ behaviors are considered, to identify potential unintended consequences of alcohol energy labelling that will benefit from being explored by future studies.

## 7 POTENTIAL FOR COMPENSATORY BEHAVIOR

The limited studies that have examined the impact of energy labelling of alcoholic drinks have examined short‐term consumer behavior (i.e., during first exposure to energy information) and therefore not examined longer‐term responses or potential unintended consequences. First, reductions to energy intake that occur due to alcohol energy labelling could theoretically be later ‘compensated’ for by consumers (e.g., by eating more later in the day) and produce no overall benefit (for energy balance). Studies examining compensatory behavior in response to food energy labelling are rare. At least two studies have found evidence that when individuals do make lower calorie choices as a result of calorie labels, some but not all of this reduction in energy is compensated for through consumption of additional energy later in the day.^78^, ^79^ However, other studies have not found evidence that later energy intake is increased after exposure to energy or nutrition labels on foods.^80^, ^81^, ^82^

It is also plausible that among a minority of people, energy information about alcoholic drinks could result in compensatory behaviors that result in an increased likelihood of experiencing alcohol related harm (e.g., restricting food intake immediately prior to or after drinking). Research sampling predominantly university students has observed a relationship between alcohol consumption and unhealthy weight control behaviors such as excessive exercise and dietary restriction^83^ and self‐induced vomiting,^84^ as a means to compensate for excess calorie intake from alcohol. This phenomenon has been colloquially labelled ‘drunkorexia’,^85^ ‘food and alcohol disturbance’,^86^ or ‘body weight conscious drinking’.^87^ In a study of college freshmen,^88^ although 14% of participants restricted calories before consuming alcohol, only 5.6% did so to avoid weight gain; the remaining students reported restricting calories to enhance alcohol's effects, which has also been shown in other studies.^89^ In a similar vein, in an experimental laboratory study which involved exposing predominantly young adult university participants to energy labelling on an alcoholic drink, participants were asked about how they might use energy labelling in future. A small minority (% not reported) of participants indicated that they would use energy labelling to try to consume more alcohol for fewer calories^90^ and in a study of US adults it was suggested that energy labelling may result in consumers falsely believing there is nutritional value in alcoholic drinks.^91^

It has also been argued that nutritional labelling could theoretically result in alcohol being more likely to be considered or treated as a food product, as opposed to being a psychoactive substance that can cause addiction and serious harm.^92^ It is important to note that not all potential compensatory behaviors in response to alcohol observed in studies are unhealthy, with some young people reporting drinking water or eating healthier foods to account for calories consumed from alcohol.^93^ Nonetheless, given that some young adults report engaging in unhealthy compensatory behaviors in response to the number of calories in alcoholic drinks, it will be important to determine whether widespread energy labelling of alcoholic drinks would increase the proportion of the general population exhibiting these types of behaviors.

## 8 POTENTIAL FOR EXACERBATION OF EATING DISORDERS

Although there is very limited evidence directly addressing the impact that energy or nutritional labelling has on individuals living with or recovering from an eating disorder, it has been argued that energy labelling may be harmful for individuals with eating disorders.^94^ In line with this argument, a survey of US college students found that close to half felt nutritional labelling of cafeteria foods could exacerbate existing eating disorders^95^ and similar concerns have been raised about energy labelling of foods in the UK.^96^ Multiple studies indicate that people who engage in unhealthy weight control practices and disordered eating are more likely to report using nutrition labels on foods,^97^, ^98^ but causality is not attributable (e.g., labels promoting disordered eating vs. disordered eating increasing likelihood of label use).

One study tested the influence of energy labelling on hypothetical food menu choices in females and measured disordered eating symptomology.^99^ When disordered eating was examined on a continuum, there was no evidence that labelling affected the energy content of hypothetical chosen meals in participants scoring higher versus lower in disordered eating. However, in a separate analysis limited by a small sample size, the presence (vs. absence) of energy labelling was associated with participants who met questionnaire cut‐offs for anorexia and bulimia nervosa symptomology selecting hypothetical meals with fewer calories and participants who met questionnaire cut‐offs for binge eating disorder selecting meals with hypothetically more calories.^99^ There was no difference observed for females without disordered eating and findings could be interpreted as indicating that energy labelling resulted in hypothetical choices consistent with maintaining eating disorder symptomology (e.g., restriction of energy in individuals with anorexia).

In a study surveying US college students before and after the introduction of energy labelling of foods in a cafeteria,^100^ there was no evidence of changes to students' body dissatisfaction, eating disturbance, anxiety or engagement in unhealthy weight control behaviors as a result of the introduction of labelling overall or in students with self‐reported eating disturbance.^100^ There are no studies examining eating disorder symptomology and the impact of energy labelling specifically related to alcohol, although a UK study found that among a small group of participants with a self‐reported eating disorder diagnosis (*n* = 23) levels of support for energy labelling of alcoholic drinks were similar to participants without an eating disorder diagnosis.^52^

At present, there is a lack of direct evidence on potential unintended consequences of energy labelling. There are also other untested ways in which energy labelling could theoretically produce undesired consequences (e.g., detracting from other warning labels on alcohol). Because the evidence reviewed here suggests it is at least plausible that energy labelling could produce undesirable outcomes in some individuals (e.g., encouraging unhealthy behavioral responses), it will be important for further research to measure whether or not energy labelling produces unintended effects and for whom. However, at present, it is important to note that there is a lack of convincing evidence that energy labelling policies produce harm.

## 9 THE IMPACT OF MANDATORY ENERGY LABELLING ON THE ALCOHOL INDUSTRY

Understanding the impact that policies requiring mandatory labelling of alcoholic drinks may have on the behavior of the alcohol industry will also be key to determining overall impacts on public health. As discussed, there was an increase in the number of alcoholic drinks products in supermarkets containing on label energy content information in the UK between 2015 and 2020, a period in which potential legislation in the EU and UK was being discussed.^101^ In 2015 energy labelling of alcoholic drinks was extremely rare with less than 5% of products containing energy labels.^21^

In 2020, an Alcohol Health Alliance (AHA) funded study found that although most beverages surveyed in UK supermarkets contained no nutritional information, energy (calorie content) information was included on 44% of sampled products.^18^ This increase is likely to be explained by a leading alcohol industry group in the UK (Diageo) announcing intentions to voluntarily include health and nutrition information on labels of leading product ranges.^102^ It is at least plausible that such actions are pre‐emptive attempts to avoid mandatory legislation,^103^ as if through voluntary practices labelling became the norm, governments may perceive there to be less of a need to pass legislation. However when energy labelling information is provided voluntarily the average height of text is substantially smaller than the recommended size for text to be easily readable,^18^ which underlines that mandatory policies and standards for energy labelling will likely be required to ensure energy labelling is fit for purpose.

There is evidence from research examining food industry responses to energy labelling requirements in the out of home food sector (e.g., restaurant menu labelling) that a small amount of product reformulation occurs when energy information has to be presented to customers at point of choice, presumably to reduce consumer concerns that products being sold are excessively unhealthy.^104^ A meta‐analysis of predominantly US studies estimated that provision of energy labelling resulted in the energy content of meals being reduced by −15 kcals on average^105^ and in the UK, restaurants and fast‐food outlets that list energy information on menus (voluntarily) tend to sell products that are lower in energy content than outlets which do not list energy information.^106^ There is also some evidence that restaurants may have removed very high calorie products from menus in response to the announcement of mandatory energy labelling requirements in the US.^107^

Similarly, there is evidence that the introduction of the soft drinks industry levy in the UK (a tax on soft drinks with more than 5 g sugar/100 ml) incentivized many manufacturers to reduce sugar in soft drinks^108^ and led to a significant reduction in the total amount of sugar sold in soft drinks in the UK.^109^ Whether the number of calories in alcoholic drinks and level of consumer concern would also motivate the alcohol industry to reformulate products to be lower in energy is unclear. However, alcohol manufacturers could reformulate products in a number of ways to reduce energy content. Reductions to both serving sizes and ABV would lower energy content. Furthermore, a 2009 US study estimated that 26% of alcoholic drink energy content is derived from non‐alcohol ingredients^110^ which would allow for further reformulation. Short‐term randomized control trials show that reductions to alcoholic drink serving size^111^ and ABV^112^ decrease amount of alcohol consumed. Therefore, reformulation could be of particular benefit to consumers as it would result in the amount of energy being consumed from alcoholic drinks decreasing without the need for conscious behavior change. If reformulation did occur and energy content was reduced by decreasing ABV, then this may also provide additional public health benefits by reducing alcohol‐related harm.

Product reformulation could result in beneficial effects to drink energy content, but it is also possible that mandatory energy labelling requirements could result in changes to alcohol industry behavior that would be detrimental to public health. Energy labelling would create costs for the alcohol industry and these costs may be attempted to be recovered through increased marketing. For example, Seltzers (carbonated water‐based low‐calorie alcoholic drinks) are a relatively new product range of alcoholic beverages in the US targeted at young adults and forecasted to increase in popularity as major corporations like Coca‐Cola launch similar product ranges in Europe.^113^ Although standardized energy labelling requirements may help consumers better understand energy content of such drinks, it is also possible that the mandatory requirement to provide energy information could result in the alcohol industry prioritizing marketing of these lower energy dense products.^114^ This could theoretically result in an increase in uptake of these products among some population sub‐groups (e.g., young adults) and if increased uptake was not directly matched with changes in preference (e.g., choosing a seltzer over a higher calorie beverage), then this could serve to increase both alcohol and energy intake in some population sub‐groups. Therefore, there are plausible alcohol industry responses to mandatory energy labelling policies that could be both beneficial or detrimental to public health and assessing industry responses to the threat or implementation of energy labelling policies will be key in future research.

## 10 FUTURE DIRECTIONS AND CONCLUSIONS

Given that the energy content of alcoholic drinks is variable but can be substantial and consumers are generally unaware of the energy content of alcoholic drinks, there is a consumer demand for the provision of energy information on alcoholic drink products. Although there are some plausible unintended consequences of alcoholic drink energy labelling, there is currently no convincing evidence that energy labelling would produce undesirable effects on health or well‐being. Because public health policies should first, do no harm, an important future research direction will be to more thoroughly study potential unintended consequences of energy labelling.

Would widespread alcohol energy labelling reduce obesity? Because of the unclear effect that alcohol has on obesity and the modest contribution calories from alcohol make to daily energy intake in the overall population, there may be limited scope for alcohol policies to substantially reduce obesity. Coupled with the observation that nutritional labelling policies produce only relatively small changes to consumer and industry behavior, energy labelling of alcoholic drinks as a standalone policy is unlikely to produce the types of effects on population level obesity as more progressive policies addressing unhealthy food advertisement, availability and price. Therefore, alcohol energy labelling policies may have some value in supplementing these types of more progressive policies, but would not be advised as a central component of any population level strategy to reduce obesity.

However, these conclusions are made with caveats. First, there is a lack of direct evidence on the impact that energy labelling of alcoholic drinks would have on both consumer behavior and the behavior of the alcohol industry and this should now be a priority for future research. Given the challenges of examining consumer behavior in the field, simulated studies (e.g., using virtual supermarket methodology or semi‐naturalistic drinking laboratory settings) could be used to generate initial evidence on consumer behavior.^115^, ^116^ These studies will also need to be supplemented with real‐world studies of alcohol energy labelling. Examples of study approaches that have examined the effect of non‐alcoholic food and drink energy labelling on consumer behavior have involved forming partnerships with retail outlets^117^ or evaluating changes to consumer behavior pre versus post implementation of labelling policies.^68^

When considering potential effects on behavior there are multiple other pathways by which alcohol energy labelling could affect energy balance and the size of their impact would not be constrained by alcohol making a modest contribution to daily energy intake (e.g., alcohol energy information encouraging increased physical activity or healthier dietary patterns). Future research and evaluations of the overall effectiveness of energy labelling will need to consider these pathways in concert and it is therefore possible that energy labelling of alcoholic drinks could make a meaningful contribution to addressing obesity as part of a collection of more extensive policies. There will also be some population groups consuming large amounts of alcohol (and calories derived from alcohol) and therefore even relatively small changes to behavior in this group as a result of energy labelling could have substantial implications on energy balance in these populations. Therefore, further research to assess how equitable across different socio‐demographic groups energy labelling of alcoholic drinks is as a public health policy to reduce obesity will be informative.

## AUTHOR CONTRIBUTIONS

All authors contributed to the study conception and design.

## CONFLICT OF INTEREST

No authors declare a direct conflict of interest. Eric Robinson has previously been the recipient of research funding from Unilever and the American Beverage Association for unrelated research.

## Supporting information

Supporting Information S1
