# Supplementary material for: Energy labelling of alcoholic drinks: An important or inconsequential obesity policy?
Source: Obes Sci Pract. 2022 Sep 23;9(2):75–86. doi: 10.1002/osp4.638 (PMC10073822; doi:10.1002/osp4.638)
Supplement: Supplementary file 1 — Supporting Information S1 [file OSP4-9-75-s001.docx]

Online Supplementary Materials

*Identification and discussion of relevant literature*

Because this was is a perspective piece as opposed to a review (i.e. we did not have a specific and predefined research question that would require identification of only studies addressing that question) we did not use systematic review methodology (e.g. standardised recording and reporting of reasons studies were not included or cited, formal extraction of data from studies). However, to inform this perspective piece we conducted multiple literature searches to identify relevant published literature for each of the different sections of the review.

For example, to inform our coverage of the contribution that alcohol has to daily calories we searched abstracts, titles, and keywords in SCOPUS and PubMed from inception to January 2021, using a combination of search terms reflecting alcohol (alcohol OR ethanol) and daily calorie intake (“daily calorie*” OR “daily energy intake” OR “daily kcal”). When exploring relevant literature for potential unintended consequences of alcohol calorie labelling we conducted similar searches of electronic databases (Medline and Scopus) and used a wide range of search terms to identify any literature that may be relevant. Example search strategy from Scopus: “( ( ( ( compensat* ) OR ( "compensat* behaviour" ) OR ( "excessive exercise" ) OR ( "physical activity" ) OR ( "skip* meals" ) OR ( "eating disorder" ) OR ( "disordered eating" ) OR ( bingeing ) OR ( purging ) OR ( "anorexia nervosa" ) OR ( bulimia ) OR ( "binge-eating disorder" ) OR ( {BED} ) OR ( "adverse effects" ) OR ( "adverse outcomes" ) OR ( "indirect effects" ) OR ( "indirect outcomes" ) ) AND ( ( ( ( calorie OR food OR menu OR alcohol* ) W/1 label* ) ) OR ( ( ( calorie OR food OR menu OR alcohol* ) W/1 info* ) ) ) )”

When considering literature, we prioritised use of existing systematic reviews and meta-analyses, randomized control trials and the most recent literature (e.g. citation of recent studies examining public acceptance of alcohol calorie labelling, as opposed to outdated studies that may no longer reflect current public opinion). Because there are some research topics that have received limited empirical attention in relation to alcohol, where appropriate we drew on research examining energy labelling of food products to consider potential mechanisms of action and consequences of energy labelling.

Given the nature of some of the literature we covered, formal academic methods for literature retrieval did not yield the required information (and early searches confirmed this). For example, potential industry responses to alcohol calorie labelling identified from industry publications or news articles on alcohol calorie labelling legislation / developments. Therefore, we supplemented database searches with a number of grey literature searches using broad search terms (e.g., alcohol + calories). These searches included key research and policy organisation websites, regulatory body websites (e.g., Advertising Standards Authority) and industry groups (e.g., the Portman Group) as well as general searches in Google (e.g., to identify news media articles on relevant trends). In addition, the main social media platforms in the UK (YouTube, Facebook, Instagram and Twitter) were searched and infographics and video commercials were viewed to observe marketing strategies that may be of relevance to alcohol calorie labelling.
